# Making it safe to speak up about futile care: a multiperspective survey on leadership, psychological safety and perceived futile care in the ICU

**DOI:** 10.1186/cc14647

**Published:** 2015-03-16

**Authors:** D Schwarzkopf, J Felfe, CS Hartog, F Bloos

**Affiliations:** 1 Jena University Hospital, Jena, Germany; 2 Helmut Schmidt University, Hamburg, Germany

## Introduction

Psychological safety (PS), for example safety of speaking up, fosters team learning and prevents treatment errors on the ICU [1]. Since speaking up might also prevent excessive and inappropriate (futile) care for patients, we hypothesized that teams with higher PS report less perceived futile care (PFC). We also expected that attending physicians' inclusive leadership (IL), which invites nurses' and residents' participation [2], would decrease PFC and that PS mediates this relationship.

## Methods

The hypotheses were tested in a cross-sectional, multicenter paper-and-pencil survey addressing medical staff on participating ICUs. A total of 22 ICUs and four intermediate care units were included in the sample and 73 attendings, 147 residents and 659 nurses participated in the study (52% participation). Psychometric properties were tested by confirmatory factor analysis (CFA), Cronbach's α and intraclass correlations (ICC). A series of hierarchical linear models (HLM) were conducted to test the study hypotheses separately among nurses/residents and attendings. IL and PS were entered as unit-level predictors (mean values per unit). Covariates were demographics, working hours per week, workload and unit size (number of staff). Mediation effects were tested.

## Results

The CFA showed a good fit indicating factorial validity (CFI: 0.97), reliabilities were from α 0.79 to 0.93 and ICCs were significant (~0.20, *P *< 0.001). HLM revealed that unit-level IL of nurses and residents was positively related to PS (*b *= 0.34, *P *< 0.001). Being a resident and working in a smaller unit also predicted PS. As expected, unit-level PS was negatively related to individual PFC (*b *= -0.38, *P *= 0.025). Further predictors of higher PFC were: being a nurse, having more than 5 years of job experience and higher workload. PS mediated the relationship between unit-level IL and individual PFC (indirect effect: -0.13, *P *< 0.001). Additional analyses revealed that attendings' PFC was negatively related to their perception of residents PS (*b *= -0.44, *P *= 0.019).

## Conclusion

A sense of PS in an ICU team might reduce futile care by increasing the safety of speaking-up behavior of nurses and residents. PS can be enhanced by attending physicians who practice inclusive leadership behavior to foster autonomy and participation of residents and nurses.
